# Trilaciclib dose selection: an integrated pharmacokinetic and pharmacodynamic analysis of preclinical data and Phase Ib/IIa studies in patients with extensive-stage small cell lung cancer

**DOI:** 10.1007/s00280-021-04239-9

**Published:** 2021-02-17

**Authors:** Chao Li, Lowell Hart, Taofeek K. Owonikoko, Raid Aljumaily, Caio Max Rocha Lima, Paul R. Conkling, Roy Timothy Webb, Robert M. Jotte, Steven Schuster, William J. Edenfield, Deborah A. Smith, Mark Sale, Patrick J. Roberts, Rajesh K. Malik, Jessica A. Sorrentino

**Affiliations:** 1 G1 Therapeutics, Inc., Research Triangle Park, NC USA; 2 Florida Cancer Specialists, SCRI, Fort Myers, FL USA; 3 Wake Forest Baptist Medical Center, Winston-Salem, NC USA; 4 Winship Cancer Institute, Emory University, Atlanta, GA USA; 5 Stephenson Cancer Center and SCRI, University of Oklahoma, Oklahoma City, OK USA; 6 US Oncology Research, Virginia Oncology Associates, Norfolk, VA USA; 7 Genesis Cancer Center, Hot Springs, AR USA; 8 Rocky Mountain Cancer Centers, Denver, CO USA; 9 Poudre Valley Health System, Fort Collins, CO USA; 10 Prisma Health Cancer Institute, Greenville, SC USA; 11 Nuventra Pharma Sciences, Durham, NC USA; 12 Present Address: Fosun Pharma USA, Inc., Lexington, MA USA; 13 Present Address: Arc Therapeutics, Research Triangle Park, NC USA

**Keywords:** CDK4/6 inhibitor, Chemotherapy-induced myelosuppression, Myelopreservation, Pharmacodynamics, Pharmacokinetics, Trilaciclib

## Abstract

**Purpose:**

Trilaciclib is a first-in-class CDK4/6 inhibitor that transiently arrests hematopoietic stem and progenitor cells (HSPCs) in the G1 phase of the cell cycle to preserve them from chemotherapy-induced damage (myelopreservation). We report integrated analyses of preclinical and clinical data that informed selection of the recommended Phase II dose (RP2D) used in trilaciclib trials in extensive-stage small cell lung cancer (ES-SCLC).

**Methods:**

A semi-mechanistic pharmacokinetic/pharmacodynamic (PK/PD) model developed from preclinical data guided selection of an optimal dose for G1 bone marrow arrest in a first-in-human Phase I study (G1T28-1-01). PK, PD, safety, and efficacy data from G1T28-1-01 and two Phase Ib/IIa studies (G1T28-02/-03) in ES-SCLC were analyzed to support RP2D selection.

**Results:**

Model simulation of bone marrow arrest based on preclinical data predicted that a ≥ 192 mg/m^2^ dose would induce a 40–50% decrease in total bone marrow proliferation in humans and almost 100% cell cycle arrest of cycling HSPCs. Consistent with this model, analysis of bone marrow aspirates in healthy volunteers after trilaciclib 192 mg/m^2^ administration demonstrated almost 100% G1 arrest in HSPCs and 40% decrease in total bone marrow proliferation, with minimal toxicity. G1T28-02/-03 reported similar PK parameters with trilaciclib 200 mg/m^2^ but slightly lower exposures than expected compared with healthy volunteers; consequently, 240 and 280 mg/m^2^ doses were also tested to match healthy volunteer exposures. Based on PK and relevant safety data, 240 mg/m^2^ was selected as the RP2D, which was also favored by myelopreservation endpoints in G1T28-02/-03.

**Conclusion:**

Integrated PK/PD, safety, and efficacy data support 240 mg/m^2^ as the RP2D for trilaciclib.

**ClinicalTrials.gov Identifiers:**

NCT02243150; NCT02499770; NCT02514447.

**Supplementary Information:**

The online version contains supplementary material available at 10.1007/s00280-021-04239-9.

## Introduction

Damage to hematopoietic stem and progenitor cells (HSPCs) by cytotoxic chemotherapy causes multilineage myelosuppression, which can manifest as neutropenia, anemia, thrombocytopenia, and/or lymphopenia [1–3]. Chemotherapy-induced myelosuppression (CIM) can lead to serious complications such as infections, bleeding, and fatigue, which frequently require hospitalization, growth factor support (granulocyte colony-stimulating factor and/or erythropoiesis-stimulating agents), and blood cell transfusions [3]. Additionally, myelosuppression often results in chemotherapy dose reductions and/or delays that limit dose intensity [1, 3, 4]. To date, there is no single treatment available that prevents or mitigates the myelosuppressive effects of chemotherapy before they occur [5]. Existing interventions are generally used reactively to treat acute cytopenias and are lineage specific; each of these has their own set of associated risks [5–9].

Trilaciclib is a first-in-class, highly potent, and selective intravenously administered cyclin-dependent kinase 4/6 (CDK4/6) inhibitor, developed to preserve multiple hematopoietic cell lineages from chemotherapy-induced damage. When administered prior to chemotherapy, trilaciclib transiently arrests CDK4/6-dependent proliferating cells in the G1 phase of the cell cycle to prevent CIM [5, 10, 11]. In preclinical studies, administration of trilaciclib prior to chemotherapy was shown to protect HSPCs from CIM, resulting in faster recovery of all blood cell lineages and mitigation of bone marrow exhaustion [10, 11]. In a Phase I first-in-human trial (Study G1T28-1-01), trilaciclib transiently inhibited bone marrow HSPC proliferation in healthy human volunteers with minimal toxicity; all moderate adverse events (AEs) spontaneously resolved within 24 h, and no AEs of severe or life-threatening intensity were reported [11, 12].

There was a theoretical concern that during dose escalation with chemotherapy, low doses of trilaciclib could produce an insufficient G1 arrest, thereby releasing HSPCs from G1 arrest in a synchronous manner while therapeutic levels of chemotherapy were present, and potentially resulting in increased CIM. Therefore, it was important to ensure that a biologically effective dose (BED) of trilaciclib was identified that was sufficient to maintain G1 cell cycle arrest of HSPCs throughout the duration of clinically relevant chemotherapy exposure. Because trilaciclib is a first-in-class molecule and no prior clinical evidence was available from similar molecules to inform dose selection, pharmacokinetic (PK) and pharmacodynamic (PD) modeling of available preclinical trilaciclib data [11, 13] was used to predict the exposure needed to induce a sufficient G1 cell cycle arrest of HSPCs in humans. In addition, the first-in-human trial in healthy volunteers (Study G1T28-1-01) was designed to evaluate exposure during dose escalation and to assess trilaciclib PD activity in the bone marrow when the predicted exposure was achieved [11, 12]. The administration of trilaciclib prior to standard-of-care chemotherapy was then evaluated in two Phase Ib/IIa trials in patients with extensive-stage small cell lung cancer (ES-SCLC): (1) Study G1T28-02 in patients with newly diagnosed ES-SCLC treated with etoposide plus carboplatin (E/P) [5]; and (2) Study G1T28-03 in patients with ES-SCLC treated with topotecan in the second-/third-line setting [14]. Both of these studies included open-label, Phase Ib dose-finding portions. The safety, PK, and efficacy data from these dose-finding portions also provided important information leading to the final dose selection for the subsequent randomized, double-blind portions of these trials, and for the randomized, double-blind, Phase II Study (G1T28-05) in patients with newly diagnosed ES-SCLC treated with E/P plus atezolizumab [15]. Across all three trials, trilaciclib showed robust myelopreservation benefits across multiple hematopoietic lineages, without negatively impacting the antitumor efficacy of chemotherapy [5, 14, 15].

Here, we describe these integrated analyses of preclinical and clinical PK, PD, safety, and efficacy data that formed the basis for selecting 240 mg/m^2^ as the dose for the Phase II randomized trials of trilaciclib in patients with ES-SCLC.

## Materials and methods

### Semi-mechanistic PK/PD modeling of preclinical data

Owing to the invasive nature of the PD marker collection (i.e. bone marrow aspiration), a semi-mechanistic PK/PD model based on animal PK/PD data and human PK data was developed to guide the selection of an optimal dose level for bone marrow aspiration in humans during dose escalation in the Phase I first-in-human study. The model was constructed using PK data from four species (mouse, rat, dog, and human) and PD data from three species (mouse, rat, and dog; Online Resources 1 and 2).

The PK/multi-compartment PD model was based on a myelosuppression model previously published by Friberg et al. [16], with modifications. Specifically, the Friberg model did not differentiate between the bone marrow stem cell and progenitor cell compartments, but combined these into a single proliferative cell compartment. In the model used in the current study, stem cells and progenitor cells were separated into two compartments, with experimental data measured for each compartment. Additionally, the current model further divided the cell cycles into G1-phase and S-phase compartments, which is critical to capture trilaciclib’s mechanism of arresting cells in the G1 phase. Another important deviation from the Friberg model was the mechanism postulated for the homeostasis of the peripheral mononuclear cells (PMNs). We postulated that the feedback mechanism has a set point target and that deviations from that target would accelerate (in the case of too few PMNs) or decelerate (in the case of too many PMNs) the transition of G1-phase cells to S-phase cells in the stem cell population.

The model was constructed in two stages. First, preclinical PK data (e.g. doses, routes, and schedule of administration, observed concentrations) were compiled and modeled by allometric scaling. A sequential PK/PD modeling approach (population PK parameters and data approach [17]) was then employed in which a semi-mechanistic PD model with various physiological compartments was created, and specific assumptions entered (Online Resource 3). Traditional forward addition/backward elimination was used for PK modeling. Forward addition/backward elimination consists of adding “features” to the model (one at a time) until the data support no more parameters, then eliminating features (one at a time) until all remaining effects are statistically significant by likelihood ratio test, or until diagnostic plots suggest that the model is less predictive. Many parameters in this complex model could not be estimated and were fixed either to the literature values or values selected on the basis of discussions with domain experts and sensitivity analysis. In preclinical animal models, the PD analysis modeled bone marrow cells positive for 5′-ethynyl-2′-deoxyuridine (EdU) and peripheral neutrophil counts simultaneously. Although the preclinical animal models could determine the G1 phase, they could not differentiate S, G2, and M phases; cells in these phases will be referred to as “cycling” throughout this manuscript.

**Fig. 1:**
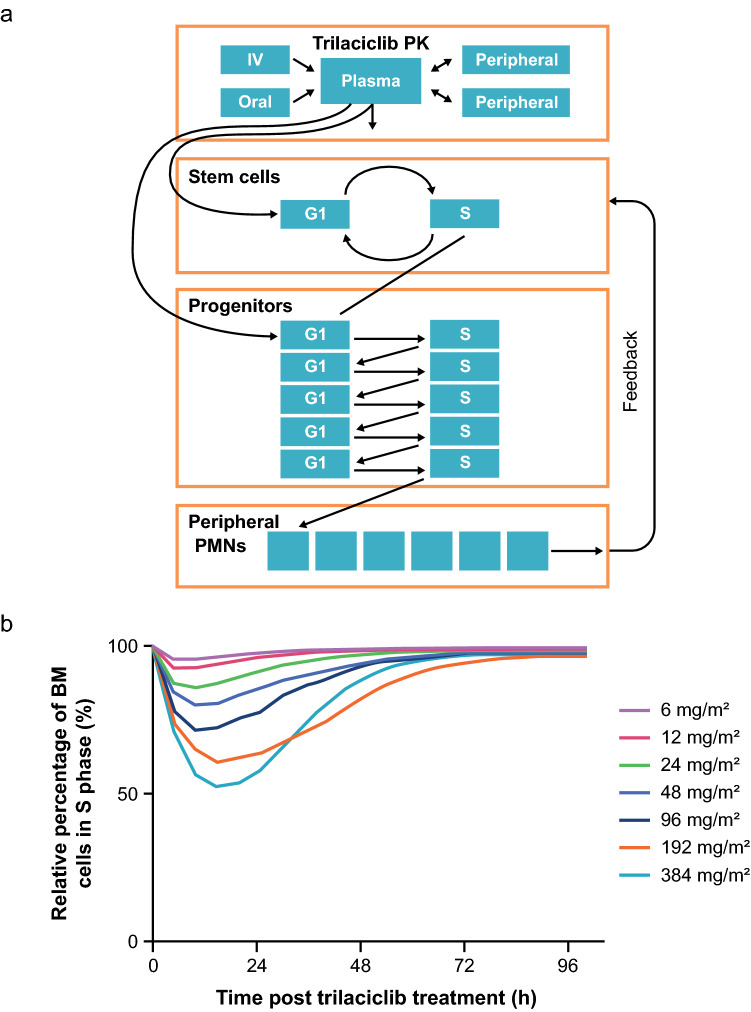
Final PK/PD model and simulation of bone marrow arrest. The final model (**a**) was a linear three-compartment model with first-order absorption. The model consisted of a population of stem cells cycling between G1 and S/G2/M phases, with a single compartment for G1 phase and a single compartment for S/G2/M phase (cycling cells). On each cycle between G1 phase and cycling, a population of bone marrow progenitor cells was created. The bone marrow model consisted of six populations of G1 phase and cycling cells arranged sequentially, such that the initial cells (from cycling stem cells into bone marrow progenitor G1 phase) progressed through five cycles of G1–S/G2/M-phase transitions. At the final bone marrow cycling phase, the population of cells transitioned to peripheral neutrophils. The peripheral neutrophil population was represented by six sequential cell populations. In addition, a feedback mechanism was modeled wherein a “target” for peripheral neutrophils was set to the initial neutrophil count and deviations from this target resulted in increased or decreased turnover of stem cells, thereby maintaining a relatively constant peripheral neutrophil count. Preclinical and clinical observations suggest there is a “persistence effect,” possibly related to change in Cyclin D, where G1 arrest is maintained for some period of time beyond trilaciclib exposure. This effect was parameterized by a slowing of the transition of S-phase stem cells back to G1 phase by the effects of trilaciclib. To represent this effect, a parameter for a “persistence effector” was incorporated, with a baseline amount set equal to the population size of S/G2/M-phase stem cells. This parameter was given an input proportional to the S/G2/M-phase stem cell population and then declined with a linear kinetics. Simulations of total bone marrow arrest (**b**) represent mean values from 500 individuals. *BM* bone marrow, *IV* intravenous, *PD* pharmacodynamics, *PK* pharmacokinetics, *PMN* polymorphonuclear cells

A combined systems biology and empiric modeling approach was taken, largely based on an understanding of the biology of hematopoiesis and the mechanism of action of trilaciclib, with literature values used when needed, and domain expert estimates used when literature values were not available. The multi-compartment PD model (Fig. 1a) was built to capture the effect of trilaciclib on hematopoiesis, with cell proliferation originating at the hematopoietic stem cell (HSC), differentiating into progenitor cells, and ultimately culminating in the release of a mature neutrophil into peripheral circulation. An additional regulatory mechanism was used to describe a “persistence effect” on the delayed transition from G1-phase stem cells to cycling stem cells even after concentrations of trilaciclib had fallen to below sub-therapeutic levels observed in preclinical studies, which may be related to the time required for the biosynthesis of required proteins, or other processes required prior to transition to the S/G2/M phase. The effect of trilaciclib was modeled as a maximal effective concentration (*E*_max_) in plasma on the transition rate constant from G1 to cycling for HSPCs. The effect of 5-fluorouracil (5-FU) was modeled as removing (killing) cells while HSPCs are cycling. Owing to model complexity, only a minority of parameters could be estimated. The remaining parameters were either fixed using literature-based values or reliance on the model to find the best fit.

Simulations were performed for total cycling bone marrow cells across different dose levels from 4 to 384 mg/m^2^. The PK model for trilaciclib was fixed to the final model from the PK analysis, and an additional 5-FU model was fixed to the literature values [18]. The literature estimate for the half-life of 5-FU is 8–20 min. The PD of 5-FU cytotoxicity, an inhibitor of thymidylate synthase [19], suggests that an indirect model [20] may be appropriate and, therefore, that PD activity may persist after the concentration has fallen. In addition, a key consideration in interpreting the result of this analysis was to prevent the release of the G1-phase cells into S phase until there was confidence that the cytotoxicity of the 5-FU was eliminated. Thus, a conservative model for 5-FU activity was sought. Neither data nor literature was available to support such a model. Therefore, a modification of the literature estimate of a half-life of 8–20 min was made, and the pharmacologic activity half-life of 5-FU was estimated at 42 min (elimination rate constant of 1/h). The 5-FU PD model then consisted of a first-order elimination model with a rate constant of 1/h. Volume could not be estimated, as no concentration data were available, and was effectively fixed to 1.0. The final PD model fixed values obtained from model fits for the transit constant for stem cells at 0.5/h (i.e. mean residence time [MRT] for cycling stem cells was estimated to be approximately 2 h; Online Resource 3). The ratio of G1-phase to cycling cells was set to 1% (stem cells spend 1% of their time cycling [21]), and the total MRT for bone marrow progenitors was set to 120 h. Under the assumption of 120 h for the total bone marrow progenitor life span (in line with reported transit time from progenitor cells to peripheral PMNs [22, 23]), the cell cycle through five G1–S/G2/M-phase cycles, with 16 h in the G1 phase and 8 h cycling per cycle, was consistent with animal data [24]. These values could not be estimated and were fixed; however, by testing feasible ranges for these parameters in the context of a sensitive model (i.e. the goodness of the model fits is sensitive to model parameters), parameter values with the best fit for the observed data were selected. In the 5-FU model, the elimination rate constant was fixed to 1/h, and the concentration resulting in 50% of maximum effect (EC_50_) for 5-FU, as identified in an earlier model (in-house modeling results), was fixed at 0.000334 ng/mL. The E_max_ for 5-FU (fraction of cells that could be killed at a very high concentration) was fixed to 1.0. In the final model, between-subject variability (log normal in all cases) was included on central volume of distribution, clearance, and the scale parameter for PMNs. Random between-species variability was included on central volume of distribution and clearance. The residual error for both bone marrow cells and PMNs was log normal.

Model diagnostics consisted of standard PK/PD plots, including basic goodness of fit, individual PK and PD predictions and observed values versus time, conditional weighted residuals versus time, and visual predictive checks (VPCs; Online Resources 4 and 5) [25].

Parameters from emerging human PK data from Phase I dose escalation were incorporated into the PK/PD model to simulate the relative percentage of total bone marrow cells cycling or arrested in the G1 phase and the relative percentage of peripheral neutrophils over time, following a single dose of trilaciclib. NONMEM version 7.3 (ICON PLC, Hanover, MD, USA) was used for parameter estimations [26]. XPOSE version 4 [27] was used for figures, and Perl-speaks-NONMEM version 3.62 [28] used for VPC calculation.

### Phase I and Phase II study designs

Details of the G1T28-1-01, G1T28-02, and G1T28-03 study designs have been reported previously [5, 11, 14].

Briefly, Study G1T28-1-01 (NCT02243150) was a single-center, single-dose, first-in-human study of trilaciclib in healthy human volunteers [11]. Healthy male and/or female subjects aged 18–60 years, with a body weight of ≥ 50 kg and a body mass index of 18–32 kg/m^2^, were enrolled. The study included a double-blind, randomized, placebo-controlled single ascending dose (SAD) part in six 4- to 8-subject cohorts (3:1; trilaciclib vs placebo [6, 12, 24, 48, 96, or 192 mg/m^2^]; 30-min intravenous [IV] infusion), and an open-label 12-subject cohort who received a single 192 mg/m^2^ dose to confirm the BED on the basis of the totality of available safety, PK, and PD data. The PD activity of trilaciclib was assessed in an ex vivo phytohemagglutinin (PHA)-stimulated lymphocyte proliferation assay (SAD and BED cohorts), and by evaluation of HSPC proliferation in bone marrow aspirates (BED cohort only). To assess the anti-proliferative activity of trilaciclib on lymphocytes, blood samples were obtained from subjects in the BED cohort and the SAD 96 and 192 mg/m^2^ cohorts pre-dose and at specified times after the end of infusion, and then treated with PHA for 48 h ex vivo to stimulate lymphocyte proliferation. Following a 1-h exposure to EdU, cells were harvested, processed, and analyzed by flow cytometry to measure the percentage of proliferation through EdU + incorporation in CD45 + /CD3 + cells (T lymphocytes). A single bone marrow aspirate at various time points relative to IV dosing of trilaciclib was obtained from all subjects enrolled in the BED cohort to determine the effect of trilaciclib on the cell cycle phases (i.e. G1 or cycling) of various bone marrow progenitor lineages, on the basis of expression of cell-surface markers. HSPC proliferation was measured before dosing (*n* = 5), 24 h after trilaciclib dosing (*n* = 3), or 32 h after trilaciclib dosing (*n* = 4), using flow cytometry [11].

G1T28-02 (NCT02499770) was a multicentre Phase Ib/IIa study in patients with newly diagnosed ES-SCLC [5]. Eligible patients were aged ≥ 18 years and had histologically or cytologically confirmed ES-SCLC, measurable disease per Response Evaluation Criteria in Solid Tumors (RECIST) version 1.1, an Eastern Cooperative Oncology Group (ECOG) performance status of 0–2, and adequate organ function. Part 1 was a Phase Ib, open-label, dose-finding portion, in which patients received trilaciclib 200 or 240 mg/m^2^, followed by Part 2, a Phase IIa, randomized, double-blind, placebo-controlled, expansion portion at the recommended Phase II dose (RP2D). All patients (Parts 1 and 2) received carboplatin (area under the concentration–time curve [AUC] 5 mg min/mL) on Day 1 and etoposide (100 mg/m^2^) on Days 1–3, and IV trilaciclib (or placebo in Part 2) was administered once daily before chemotherapy. The primary objective of the study was to define the trilaciclib RP2D (Part 1) and to assess the safety and tolerability of trilaciclib in combination with E/P (Parts 1 and 2). Secondary objectives included analysis of the PK profiles of trilaciclib and of E/P (Part 1), and assessment of trilaciclib efficacy (Parts 1 and 2), including thorough evaluation of common myelosuppression endpoints. In the dose-finding Part 1 portion, blood samples for PK analysis were collected at prespecified time points on Cycle 1 Day 1 and Cycle 1 Day 3 (200 mg/m^2^ cohort: all patients; 240 mg/m^2^ cohort: optional).

G1T28-03 (NCT02514447) was a multicentre Phase Ib/IIa study in patients with previously treated ES-SCLC [14]. Patients were aged ≥ 18 years and had histologically or cytologically confirmed ES-SCLC that had progressed during or after prior first- or second-line chemotherapy, measurable disease per RECIST version 1.1, an ECOG performance status of 0–2, and adequate organ function*.* Part 1 was a Phase Ib, open-label, dose-finding portion. Part 2 was a Phase IIa, randomized, double-blind, placebo-controlled portion*.* In Part 1, trilaciclib (200, 240, or 280 mg/m^2^) was administered once daily prior to chemotherapy on Days 1–5 of each 21-day topotecan cycle (IV topotecan [0.75, 1.0, 1.25, or 1.5 mg/m^2^]; Days 1–5). In Part 2a, patients were randomized 2:1 to receive trilaciclib 240 mg/m^2^ prior to topotecan 0.75 mg/m^2^, or placebo prior to topotecan 1.5 mg/m^2^ using the same schedule as that in Part 1. Part 2b evaluated trilaciclib 240 mg/m^2^ prior to topotecan 1.5 mg/m^2^ using the same schedule as that in Part 1. Study objectives included assessment of dose-limiting toxicities (DLTs; Part 1), safety and tolerability (Parts 1 and 2), PK (mainly Part 1), and myelosuppression efficacy (Parts 1 and 2) of trilaciclib in combination with topotecan. In Part 1 of the study, blood samples for PK analysis were collected at prespecified time points on Cycle 1 Day 1 and Cycle 1 Day 4 for all patients.

All studies were designed and conducted in compliance with the principles of the Declaration of Helsinki and the Good Clinical Practice guideline of the International Council for Harmonisation. Study protocols and study-related materials were approved by the institutional review board or independent ethics committee of each investigational site. Written informed consent was obtained from each patient before the initiation of study procedures.

## Results

### Interpretation of preclinical data

Previously reported preclinical data and PK/PD modeling were used to estimate the dose level at which a maximal decrease in the percentage of cycling HSCs would be expected, to be further assessed using bone marrow sampling in healthy human volunteers. Data obtained using murine HSCs indicated that a single dose of trilaciclib induced a dose-dependent, reversible cell cycle arrest of all hematopoietic cell types, and that concentrations resulting in the maximum (~ 50%) decrease from baseline in the relative percentage of total bone marrow cycling cells were effective in decreasing the percentage of cycling HSPCs so that almost 100% of HSPCs were arrested in the G1 phase [11]. Similar dose-dependent effects on G1 arrest of total bone marrow cycling cells (~ 50% maximum decrease from baseline) were observed in dogs [13], although results on specific cell types were not available owing to study limitations. Since hematopoietic development is well-conserved across species, trilaciclib’s CDK4/6 inhibition in humans was expected to be similar to that in mice and dogs, and mouse and dog data infer that a dose level resulting in an approximate 50% decrease in total bone marrow cycling cells would result in an almost 100% decrease in the percentage of cycling HSPCs, representing a therapeutically effective dose.

### PK/PD model

The final PD model (Fig. 1a) suggested an EC_50_ value of 5.9 ng/mL for trilaciclib, which predicts an EC_90_ (therapeutically active dose) of approximately 50 ng/mL. Model simulation of bone marrow arrest predicted that a trilaciclib dose of ≥ 192 mg/m^2^ would induce a 40–50% decrease from baseline in total bone marrow proliferation, with a return to baseline at approximately 48 h (Fig. 1b). The level of decrease was close to the maximum effect observed in animal studies. In addition, per simulation, a higher dose of 384 mg/m^2^ showed a similar PD effect. Therefore, 192 mg/m^2^ was selected as the dose to obtain bone marrow aspirates from healthy subjects enrolled in the first-in-human study (G1T28-1-01).

### First-in-human trial in healthy volunteers (study G1T28-1-01)

Of the 33 subjects enrolled in the SAD part of the study, 24 received trilaciclib (6 mg/m^2^, *n* = 3; 12 mg/m^2^, *n* = 3; 24 mg/m^2^, *n* = 3; 48 mg/m^2^, *n* = 3; 96 mg/m^2^, *n* = 6; 192 mg/m^2^, *n* = 6), and nine received placebo. Twelve additional subjects, who were enrolled to confirm the BED, received trilaciclib at a dose of 192 mg/m^2^. Demographic characteristics, published previously [11], were comparable among the seven trilaciclib groups and the placebo group. Geometric mean (gMean) maximum plasma concentration (C_max_)and total systemic exposure [AUC from time 0 to infinity (AUC_0-inf_)] increased dose dependently following a single 30-min IV infusion of trilaciclib over the dose ranges tested (0–192 mg/m^2^), with AUC_0-inf_ increasing slightly more than dose proportionally. Mean (range) AUC_0-inf_ for both 192 mg/m^2^ groups combined (*n* = 18) was 3029 (2379–3869) ng h/mL (Online Resource 6).

**Fig. 2:**
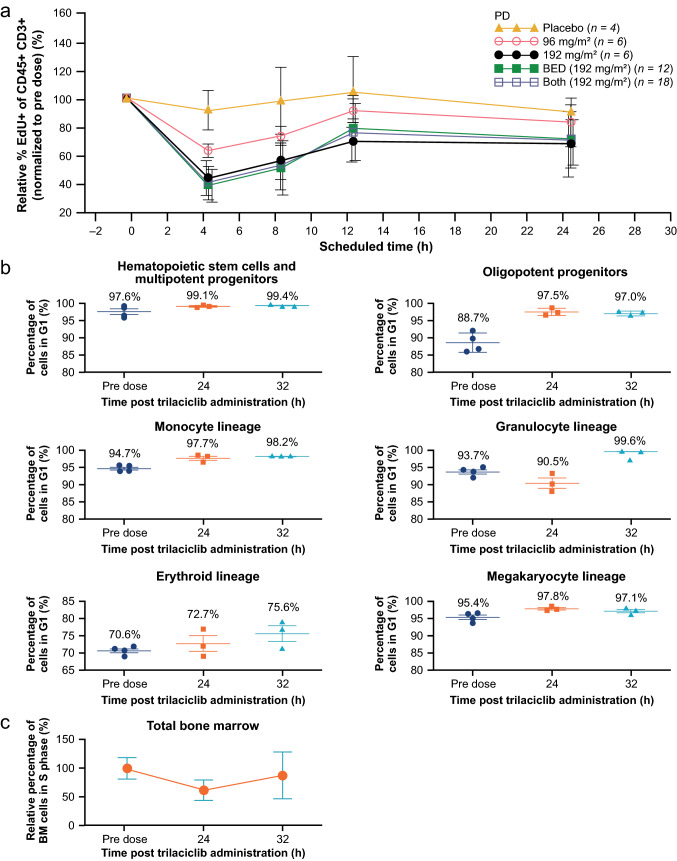
Effect of trilaciclib on lymphocyte and human bone marrow proliferation. **a** Relative percentage of EdU+ cells of the CD45+/CD3+ lymphocyte population assessed in an ex vivo (PHA)-stimulated lymphocyte proliferation assay. Blood samples were drawn from subjects in the SAD (96 and 192 mg/m^2^) and BED cohorts pre-dose and at 4, 8, 12, and 24 h after the end of infusion, and then treated with PHA for 48 h to stimulate lymphocyte proliferation. **b** Percentage of bone marrow progenitor subsets in the G1 phase at 24 and 32 h post trilaciclib dose. Bone marrow aspirates were drawn from subjects in the BED cohort at various time points [pre-dose (*n* = 5), and 24 (*n* = 3) or 32 (*n* = 4) h post trilaciclib dose]. Two bone marrow samples (one pre-dose and one at 32 h post-dose) were excluded from analysis owing to contamination with peripheral blood. **c** Relative percentage of total bone marrow cells in the S phase at 24 and 32 h post-trilaciclib dose (*n* = 10). Data shown are mean ± standard deviation. *BED* biologically effective dose, *BM* bone marrow, *EdU* 5′-ethynyl-2′-deoxyuridine, *PD* pharmacodynamics, *PHA* phytohemagglutinin, *SAD* single ascending dose

At doses of 96 and 192 mg/m^2^, trilaciclib demonstrated dose-dependent inhibition of the proliferation of CD45 + /CD3 + lymphocytes (a surrogate of CDK4/6 inhibition in hematopoietic cells) with a maximum mean inhibition of 37.2% and 60%, respectively, at 4 h after end of infusion (Fig. 2a). This indicated that the 96 mg/m^2^ dose was on the ascending part of the dose–response curve and the percentage of inhibition was suboptimal. Peripheral lymphocyte proliferation started to recover from 8 h after end of infusion; however, the variability makes it hard to conclude if it has fully recovered at the last sampling time point 24 h after end of infusion. In bone marrow aspirates collected following infusion of 192 mg/m^2^ trilaciclib in the BED cohort, a clear increase in the percentage of cells in G1 arrest was observed at 24 and 32 h post-dose for most bone marrow progenitor subsets (Fig. 2b). For HSPCs, the percentage of cells in the G1 cell cycle phase increased over time, with a maximum increase observed at 32 h post-dose. At 192 mg/m^2^, trilaciclib achieved almost 100% G1 arrest for most of the cell types, with the exception of erythrocyte and megakaryocyte lineages (Fig. 2b). By comparison, analysis of total bone marrow demonstrated an approximate 40% arrest at 24 h, with partial recovery at 32 h (Fig. 2c), which was in line with the level of decrease in total cycling bone marrow cells and cycling HSPC observed in the preclinical studies. In addition, it was consistent with the PK/PD model predictions. Given that the arrest for most of the cell types reached almost 100%, it suggests that the 192 mg/m^2^ dose reached the plateau part of the dose–response curve. Thus, it was assumed that a further increase in the trilaciclib dose would result in a diminishing return for efficacy and could result in deeper and more prolonged neutropenia.

### Phase Ib/IIa studies in ES-SCLC

#### Study G1T28-02

**Fig. 3:**
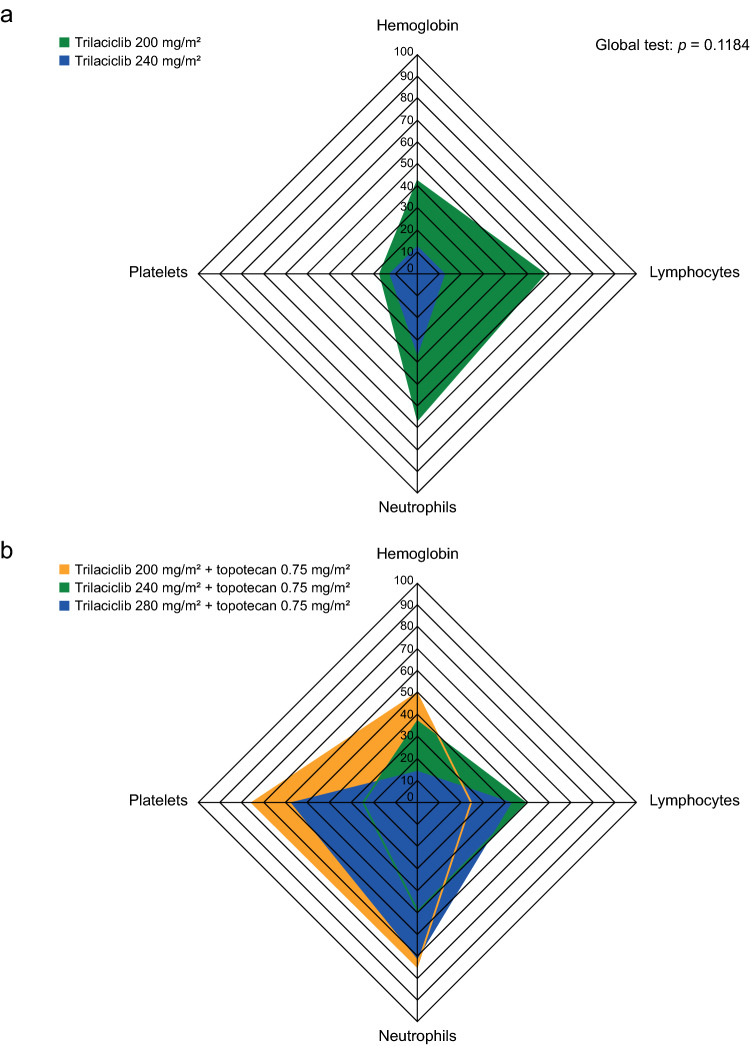
Radar plots of Grade 3/4 laboratory abnormalities in **a** Part 1 of Study G1T28-02 [Safety Analysis Set, all enrolled patients who received at least one dose of any study drug (*n* = 20)] and **b** Part 1 of Study G1T28-03 [Intent-to-Treat Analysis Set, all enrolled patients who received at least one dose of any study drug (*n* = 32)]. The radar charts provide a graphic visualization of the efficacy of each trilaciclib dose for the two studies. The charts display Grade 3/4 laboratory abnormalities for hemoglobin, lymphocytes, neutrophils, and platelets to create a unique shape for each treatment group. Each axis of the chart represents the proportion of Grade 3/4 abnormalities for a hematology laboratory parameter, with proportion increasing towards the vertex of the axis. The shaded area of the whole shape, reflecting the multi-lineage Grade 3/4 abnormalities, was compared between treatment groups using a multivariate test to generate the *p *value

In Study G1T28-02, a total of 94 patients received study drug per protocol (Part 1: trilaciclib 200 mg/m^2^ + E/P, *n* = 10; trilaciclib 240 mg/m^2^ + E/P, *n* = 9; Part 2: placebo + E/P, *n* = 37; trilaciclib 240 mg/m^2^ + E/P, *n* = 38). Baseline demographics and disease characteristics were generally comparable across the treatment groups in Part 1 [5]. PK analysis in Part 1 (*n* = 9) demonstrated an AUC_0-inf_ of 2560 (range 1390–3730) ngh/mL on Cycle 1 Day 1 in the trilaciclib 200 mg/m^2^ cohort (Online Resource 6), which was lower than the target AUC (target: 3100 ngh/mL) established in Study G1T28-1-01 at 192 mg/m^2^. On the basis of these observations and relevant safety data, the safety monitoring committee decided to increase the dose of trilaciclib to 240 mg/m^2^ to match the exposure. DLT evaluation also supported the selection of trilaciclib 240 mg/m^2^ as the Phase IIa dose. Two DLTs were reported with trilaciclib 200 mg/m^2^ (Grade 4 thrombocytopenia, and Grade 2 neutropenia resulting in Cycle 2 delay) and one with trilaciclib 240 mg/m^2^ (Grade 2 neutropenia resulting in Cycle 2 delay). Evaluation of hematology data from Part 1 by cohort and trilaciclib dose level identified a consistent reduction in Grade 3/4 laboratory abnormalities for hemoglobin, lymphocytes, neutrophils, and platelets (Fig. 3a) and multiple myelosuppression endpoints [duration of severe neutropenia (DSN) and occurrences of severe neutropenia (SN), red blood cell (RBC) transfusions, granulocyte colony-stimulating factor administration, platelet transfusions, erythropoietin-stimulating agent administration, IV antibiotic use, infection serious AEs (SAEs), and pulmonary infection SAEs; Online Resource 7] for the 240 mg/m^2^ dose compared with the 200 mg/m^2^ dose, further supporting the selection of 240 mg/m^2^ as the RP2D for trilaciclib.

#### Study G1T28-03

Thirty-two patients received study treatment in Part 1 of Study G1T28-03 [14], across seven dose cohorts (Cohort 1: trilaciclib 200 mg/m^2^ + topotecan 1.5 mg/m^2^, *n* = 2; Cohort 2: trilaciclib 200 mg/m^2^ + topotecan 1.25 mg/m^2^, *n* = 3; Cohort 3: trilaciclib 200 mg/m^2^ + topotecan 0.75 mg/m^2^, *n* = 4; Cohorts 4 and 6: trilaciclib 240 mg/m^2^ + topotecan 0.75 mg/m^2^, *n* = 8; Cohort 5: trilaciclib 280 mg/m^2^ + topotecan 0.75 mg/m2, *n* = 7; Cohort 7: trilaciclib 240 mg/m^2^ + topotecan 1 mg/m^2^, *n* = 8). In general, demographics were similar for all treatment groups in Part 1. In Part 2, 90 patients were randomized and received at least one dose of study drug (placebo + topotecan 1.5 mg/m^2^, *n* = 28; trilaciclib 240 mg/m^2^ + topotecan 0.75 mg/m^2^, *n* = 30; trilaciclib 240 mg/m^2^ + topotecan 1.5 mg/m^2^, *n* = 32).

Similar to Study G1T28-02, PK analysis (*n* = 31) showed that the AUC_0-inf_ of the 200 mg/m^2^ dose was lower [gMean (range) 2410 (1700–3000) ng h/mL on Day 1] than expected on the basis of exposure at 192 mg/m^2^ in Study G1T28-1-01; this prompted the safety monitoring committee to increase the dose to 240 mg/m^2^, which almost achieved the target AUC of 3100 ng h/mL (2910 and 2880 h ng/mL on Days 1 and 4, respectively). A dose of 280 mg/m^2^ was also tested, but exposures were significantly higher and resulted in drug accumulation (gMean AUC: 3490 h ng/mL on Day 1 and 4520 h ng/mL on Day 4) that had not been observed at lower dose levels. The safety and efficacy of three dose levels of trilaciclib: 200, 240, and 280 mg/m^2^ were evaluated when trilaciclib was administered prior to 0.75 mg/m^2^ of topotecan. No DLT (0 of 10 patients) was reported for the 240 mg/m^2^ trilaciclib cohorts. By contrast, 2 of 4 and 2 of 7 patients reported DLTs in the 200 mg/m^2^ and 280 mg/m^2^ trilaciclib cohorts, respectively. From laboratory abnormalities, the 240 mg/m^2^ dose showed fewer Grade 3/4 neutropenia and thrombocytopenia events than did the 200 and 280 mg/m^2^ doses (Fig. 3b). In addition, the 240 mg/m^2^ dose also showed improved myelopreservation compared with the 200 and 280 mg/m^2^ doses, on the basis of the primary efficacy endpoints of DSN and occurrence of SN, as well as several key secondary and supportive secondary myelopreservation endpoints (occurrences of RBC and platelet transfusions, IV antibiotic use, infection SAEs, pulmonary infection SAEs, and febrile neutropenia, and incidence of major adverse hematologic events; Online Resource 7). The efficacy/safety comparisons for trilaciclib dose levels were based on a topotecan dose of 0.75 mg/m^2^, which is half of the recommended dose (1.5 mg/m^2^). However, the PD effect of trilaciclib is assumed to be related to host cells, hence is independent of the chemotherapy dose.

## Discussion

To rationally design a schedule for administering trilaciclib prior to chemotherapy, it was critical to understand the magnitude and duration of G1 cell cycle arrest of HSPCs at a given dose level of trilaciclib. Therefore, in addition to obtaining first-in-human safety experience, a key goal of the Phase I study was to demonstrate the biological activity of trilaciclib, including the maximal level of inhibition and the duration of response in HSPCs, and to define the starting dose level of trilaciclib for use in combination with chemotherapy in patients with ES-SCLC. Owing to the difficulty of sampling human bone marrow for HSPCs and other cell types, dose selection was started from an evaluation of available preclinical data from mice and dogs [11, 13], which suggested that an approximate 50% decrease from baseline in total bone marrow cycling cells would translate into nearly 100% of HSPCs being arrested in G1 phase. Mechanistic PK/PD modeling predicted that a dose of 192 mg/m^2^ would result in a decrease in total bone marrow cycling cells of slightly less than 50%; therefore, this dose was selected as the BED, to be evaluated further in the first-in-human Phase I study (G1T28-1-01).

Lymphocytes are known to be sensitive to CDK4/6 inhibition and were evaluated as a surrogate PD marker of trilaciclib activity as well as a surrogate for bone marrow aspirates. In healthy volunteers, a robust PD effect was demonstrated with trilaciclib 96 mg/m^2^ and 192 mg/m^2^, with a dose-dependent decrease in PHA-stimulated lymphocyte proliferation, providing proof of concept that there was a dose-dependent decrease in proliferating CDK4/6-dependent cells in the blood due to CDK4/6 inhibition. In addition, the significantly higher level of inhibition for lymphocyte proliferation at 192 mg/m^2^ aligned with the simulation results that supported 192 mg/m^2^ as the BED for assessing bone marrow proliferation. Data from bone marrow HSPCs were more definitive, providing information for the target organ and cell populations. These data demonstrated that administration of trilaciclib at the BED of 192 mg/m^2^ resulted in almost 100% G1 arrest in most bone marrow stem and progenitor subsets, with the exception of erythrocyte and megakaryocyte lineages, which persisted for 32 h.

The total bone marrow data were consistent with the model-predicted results, showing an approximate 40% decrease in total bone marrow cells in the S phase with some recovery by 32 h. Trilaciclib was well tolerated at all test doses. The most commonly reported AEs were headache and nausea. No severe AEs were reported, and all moderate AEs spontaneously resolved within 24 h [11].

On the basis of PK/PD data from Study G1T28-1-01, which indicated that trilaciclib 192 mg/m^2^ was pharmacologically active with minimal toxicity in healthy volunteers, a dose of trilaciclib 200 mg/m^2^ (rounded up from 192 mg/m^2^) was selected as the starting dose for the subsequent Phase Ib/IIa ES-SCLC studies. Subsequent PK assessments across Part 1 of Studies G1T28-02 and G1T28-03 revealed that the PK parameters of trilaciclib at 200 mg/m^2^ were similar in each study, but that exposures (AUC) were slightly lower than the target AUC established with 192 mg/m^2^ in Study G1T28-1-01. On the basis of these observations and relevant safety data, the dose of trilaciclib was increased to 240 mg/m^2^. Trilaciclib was also tested at a dose of 280 mg/m^2^ in Study G1T28-03; however, owing to the level of accumulation and accompanying safety data, the 280 mg/m^2^ dose was not selected as the RP2D. The poorer efficacy observed in myelosuppression endpoints with the 280 mg/m^2^ dose may be due to the longer-than-necessary arrest of HSPCs in the G1 phase. This likely resembles an inverted U-shaped dose–response curve, whereby prolonged inhibition could cause myelosuppression.

Evaluation of safety data in Part 1 of both the G1T28-02 and G1T28-03 studies supported the selection of trilaciclib 240 mg/m^2^ as the Phase IIa dose. DLT assessments showed reduced DLT with trilaciclib 240 mg/m^2^ versus 200 mg/m^2^ (G1T28-02 and G1T28-03) and 280 mg/m^2^ (G1T28-03). Additionally, there was a reduction in hematologic Grade 3/4 laboratory abnormalities with the 240 mg/m^2^ dose compared with the 200 mg/m^2^ (G1T28-02 and G1T28-03) and 280 mg/m^2^ (G1T28-03) doses.

Efficacy data from Part 1 of both the G1T28-02 and G1T28-03 trials clearly showed that trilaciclib 240 mg/m^2^ reduced the risk of CIM in patients with ES-SCLC undergoing chemotherapy, and that it had a favorable outcome over the 200 mg/m^2^ dose. These findings are consistent with the subsequent Phase IIa efficacy results of the G1T28-02 and G1T28-03 studies, which demonstrated a clinically and statistically significant reduction in the DSN in Cycle 1 and occurrence of SN across the treatment period when trilaciclib was administered prior to chemotherapy [5, 14]. In addition to informing dose selection, the PK/PD model provided important information regarding the optimal timing of trilaciclib administration relative to chemotherapy. The duration of cycling stem cells was estimated to be in the order of 1.5–2 h, suggesting that, once trilaciclib has reached its therapeutic concentration (EC_90_ ~ 50 ng/mL), cells in the S, G2, and M phases would be fully depleted (and, therefore, protected from cytotoxicity) in approximately 1.5–2 h. Even shorter periods of trilaciclib administration prior to chemotherapy will still result in substantial protection, particularly for stem cells, as only a small fraction of stem cells are cycling at any one time [11, 29]. More important than arresting all HSPCs in the G1 phase prior to chemotherapy administration is ensuring that the therapeutic effect of trilaciclib persists throughout the duration of cytotoxic exposure, because releasing HSPCs from G1 arrest in a synchronous manner could lead to significant myelosuppression.

In summary, a PK/PD model was constructed to predict human PD of trilaciclib for the first-in-human study to guide selection of the optimal dose at which to evaluate bone marrow aspirates, which is an invasive procedure. Using model simulations and available PK and PD data from Study G1T28-1-01, a trilaciclib dose of 192 mg/m^2^ was identified as the BED and was selected for further safety and efficacy evaluation. The prediction for the PD effect estimated from preclinical data was confirmed by analyses of bone marrow aspiration from the first-in-human study. Subsequent evaluation of trilaciclib administered prior to chemotherapy (E/P or topotecan) in patients with ES-SCLC led to a PK- and efficacy-guided dose adjustment with the selection of trilaciclib 240 mg/m^2^ as the RP2D for Part 2 of Studies G1T28-02 and G1T28-03. In Parts 1 and 2 of both studies, trilaciclib 240 mg/m^2^ was well tolerated, with few trilaciclib-related AEs, and demonstrated a decrease in laboratory abnormalities and additional myelosuppression endpoints compared with placebo. Improvements were consistently more favorable in patients treated with trilaciclib 240 mg/m^2^ compared with those receiving trilaciclib 200 mg/m^2^ or 280 mg/m^2^, supporting trilaciclib 240 mg/m^2^ as the RP2D.

## Supplementary Information

Below is the link to the electronic supplementary material.

Supplementary file1 (DOCX 330 KB)

## Data Availability

All data relevant to the study are included in the article or uploaded as online supplemental information.
