# Supplementary material for: Trilaciclib dose selection: an integrated pharmacokinetic and pharmacodynamic analysis of preclinical data and Phase Ib/IIa studies in patients with extensive-stage small cell lung cancer
Source: Cancer Chemother Pharmacol. 2021 Feb 17;87(5):689–700. doi: 10.1007/s00280-021-04239-9 (PMC8026479; doi:10.1007/s00280-021-04239-9)
Supplement: Supplementary file 1 — Supplementary file1 (DOCX 330 KB) [file 280_2021_4239_MOESM1_ESM.pdf]

Supplementary material to:

Trilaciclib dose selection: an integrated pharmacokinetic and pharmacodynamic analysis of preclinical data and Phase Ib/IIa studies in patients with extensive-stage small cell lung cancer

Chao Li[1,12] • Lowell Hart[2,3] • Taofeek K. Owonikoko[4] • Raid Aljumaily[5] • Caio Max Rocha Lima[3] • Paul R. Conkling[6] • Roy Timothy Webb[7] • Robert M. Jotte[8] • Steven Schuster[9] • William J. Edenfield[10] • Deborah A. Smith[11] • Mark Sale[11] • Patrick J. Roberts[1,13] • Rajesh K. Malik[1] • Jessica A. Sorrentino[1]

Affiliations

[1] G1 Therapeutics, Inc., Research Triangle Park, NC, USA

[2] Florida Cancer Specialists, SCRI, Fort Myers, FL, USA

[3] Wake Forest Baptist Medical Center, Winston-Salem, NC, USA

[4] Winship Cancer Institute, Emory University, Atlanta, GA, USA

[5] Stephenson Cancer Center and SCRI, University of Oklahoma, Oklahoma City, OK, USA

[6] US Oncology Research, Virginia Oncology Associates, Norfolk, VA, USA

[7] Genesis Cancer Center, Hot Springs, AR, USA

[8] Rocky Mountain Cancer Centers, Denver, CO, USA

[9] Poudre Valley Health System, Fort Collins, CO, USA

[10] Prisma Health Cancer Institute, Greenville, SC, USA

[11] Nuventra Pharma Sciences, Durham, NC, USA

[12]Present Address: Fosun Pharma USA, Inc., Lexington, MA, USA.

[13]Present Address: Arc Therapeutics, Research Triangle Park, NC, USA.

Corresponding author: Rajesh K. Malik, MD; 700 Park Offices Drive, Suite 200, Research Triangle Park, NC 27709, USA; E-mail: rmalik@g1therapeutics.com

Journal: Cancer Chemotherapy and Pharmacology

Online Resource 1 List of studies used in PK and PD models

Data set	Species	Administration route	Endpoint
1	Rat	IV	PK
2	Dog	IV	PK
3	Dog	IV	PK
4	Mouse	Oral	PK
5	Dog	IV	PK
6	Dog	IV	PK; BM/EdU
7	Mouse	IP	PK
8 (Study G1T28-1-01)	Human	IV	PK
9	Mouse	Oral	BM/EdU
10	Mouse	IP	PMNs
11	Mouse	IP	PMNs
12	Mouse	IP	BM/EdU
13	Rat	IV	PMNs
14	Dog	Oral	PMNs
15	Dog	IV	BM/EdU

BM bone marrow, *EdU* 5'-ethynyl-2'-deoxyuridine, *IP* intraperitoneal, *IV* intravenous, *PD* pharmacodynamics, *PK* pharmacokinetics, *PMN* polymorphonuclear cell

Online Resource 2 Brief description of animal studies used in PK and PD models

Preclinical pharmacokinetic (PK)/toxicokinetic studies were conducted in mice, rats, and dogs. Data from eight studies in four species, with a total of 1628 observations from 191 individuals, were used for PK/pharmacodynamic (PD) modeling. Administration routes included oral, intravenous (IV), and intraperitoneal (IP). The designs of these studies are described briefly below:

- In mice, trilaciclib was given as a single dose of 150 mg/kg orally, or 50 or 100 mg/kg via IP infusion. PK samples were collected from pre dose up to 48 h post dose. In rat toxicology studies, rats received 7-day repeated doses of trilaciclib at 10, 50, or 150 mg/kg via 30-min IV infusion. PK samples were collected from pre dose up to 24 h post dose on Day 1 and Day 7.
- In dog PK studies, beagle dogs received a single 30-min IV infusion of 10, 25, or 62.5 mg/kg trilaciclib. PK samples were collected from pre dose up to 30 h post dose.
- In dog toxicology studies, dogs received 7-day repeated doses of trilaciclib at 5, 15, or 45 mg/kg via 30-min IV infusion. PK samples were collected from pre dose up to 24 h post dose on Day 1 and up to 30 h post dose on Day 7.

PD endpoints were measured in multiple mouse, rat, and dog studies. Seven hundred two observations from 466 individuals across these three species were used for the analysis. The designs of these studies are described briefly below:

- To evaluate the effect of trilaciclib on the proliferation of different mouse hematopoietic cells, mice were given a single IP dose of trilaciclib at 150 mg/kg, followed 30 min later by a single IP injection of 250 µg 5'-ethynyl-2'-deoxyuridine (EdU) to label cells in the S phase of the cell cycle. Two hours after EdU injection, bone marrow (BM) cells were harvested and analyzed for cell frequency and EdU incorporation by flow cytometry. EdU incorporation in whole BM, hematopoietic stem cells (L-S+K+CD150+CD48-), multipotent progenitors (Lin-Sca+cKit+), oligopotent progenitors (Lin-cKit+), monocytic lineage (Mac1+Gr1+), erythrocytes (Ter119+), and B cells (B220+) was evaluated. To determine the ability of trilaciclib to modulate chemotherapy-induced myelosuppression, mice were given single oral doses of trilaciclib at 150 mg/kg, followed 30 min later by a single IP dose of 5-fluorouracil (5-FU) at 150 mg/kg. Complete blood counts were performed on Days 6, 8, 10, 12, 14, and 16 after treatment.
- In dog PD studies, dogs received a 30-min infusion of trilaciclib at 1, 5, and 15 mg/kg, and BM samples were collected at approximately 8, 16, and 24 h after the infusion ended. Approximately 2 h before sample collection, each dog was given a single 10-mg/kg IV dose of EdU as a slow bolus injection. Blood samples were collected before dosing and at 24, 48, 72, 168, 240, and 336 h post dose for hematology analyses. Changes in BM proliferation after trilaciclib treatment were determined by measuring the frequency of EdU+ cells and the intensity of the EdU population. DAPI stain was used as a counterstain for cell cycle analysis, and data were analyzed by flow cytometry.

Polymorphonuclear cells were measured in the 7-day toxicology studies in rats and IP PK studies in mice mentioned above, as well as in a 14-day repeat dose toxicology study in dogs, in which dogs received 15 and 45 mg/kg trilaciclib orally.

Online Resource 3 PK/PD model parameters and assumptions

	Values	Source
PK model parameters for trilaciclib[a]		
V (L/kg)	1.32	Estimated
K_a (/h)	20.0 (FIXED)	Assumption[b]
CL (L/kg*h)	2.77	Estimated
Bioavailability (oral, mouse)	0.62	Estimated
Intercompartment rate constant K23 (/h)	15.94	Estimated
Intercompartment clearance K32 (/h)	4.42	Estimated
Intercompartment clearance K24 (/h)	2.03	Estimated
Intercompartment clearance K42 (/h)	0.29	Estimated
Allometric exponent for V	1.00	Estimated
Allometric exponent for CL	1.00 (FIXED)	Assumption[c]
Additive error	2.00 (FIXED)	Assumption[d]
Proportional error	0.32	Estimated
PD model parameters for trilaciclib and 5-FU		
Rate constant for BM precursor G to S/G2/M phase (/h)	0.04 (FIXED)	Assumption[e]
Rate constant for neutrophils transit in in periphery	$3.24*E^{-2}$ (FIXED)	Assumption[f]
Concentration of 5-FU with 50% max effect (ng/mL)	$3.34*E^{-4}$	Estimated
Max effect of G1T28 in the E_{max} model	1.00 (FIXED)	Assumption[g]
Concentration of G1T28 with 50% max effect (ng/mL)	5.90	Estimated
Feedback slope (/h)	$1.00*E^{-6}$ (FIXED)	Assumption[h]
Scale for BM in mice	1270	Estimated
Scale for neutrophils in mice	150	Estimated
Scale for neutrophils in dogs	848	Estimated

EC_{50} as fraction of baseline value of persistence effect. Effect is on transition of S phase cells to G1	2.00	Estimated
Rate constant for stem cell G to S/G2/M phase (/h)	0.51	Estimated
Residual error variance	0.73	Estimated

[a]PK model parameters for 5-FU were obtained from the literature

[b]The absorption for trilaciclib was very fast following extra-vascular administration. K_a could not be estimated and was fixed to a large value of 20, corresponding to a T_{max} of approximately 10 min

[c]The allometric exponent for CL was estimated to be 0.945 from an earlier model during the model development process and was fixed to 1.00 for the purpose of model simplification to avoid overparameterization

[d]The additive error was fixed to 20% of the assay LLOQ (10 ng/mL) to decrease the number of parameters to fit

[e]The rate constant for transiting from G phase to S phase could not be estimated. The parameter was fixed to 0.04, which corresponds to an MRT of 25 h and a life span of approximately 5 days. This is consistent with published data showing the lifespan of RBC precursors in the BM to be approximately 5 days: Pérez-Ruixo JJ, Krzyzanski W, Hing J (2008) Pharmacodynamic analysis of recombinant human erythropoietin effect on reticulocyte production rate and age distribution in healthy subjects. Clin Pharmacokinet 47 (6):399-415. https://dx.doi.org/10.2165%2F00003088-200847060-00004

[f]The peripheral neutrophil rate constant was fixed to 0.0324/h, representing a mean residence time of 31 h per compartment and a total life span of neutrophils of approximately 185 h (7.7 days). This is consistent with published data of 5.4 days: Pillay J, den Braber I, Vrisekoop N, Kwast LM, de Boer RJ, Borghans JAM, Tesselaar K, Koenderman L (2010) In vivo labeling with $^{2}H_2O$ reveals a human neutrophil lifespan of 5.4 days. Blood 116 (4):625-627. https://doi.org/10.1182/blood-2010-01-259028

[g]The model assumes that, at very high concentration, trilaciclib can achieve 100% inhibition with full arrest

[h]Based on testing a range of values to evaluate the model fit

5-FU 5-fluorouracil, *BM* bone marrow, *CL* clearance, E_{max} maximum effect, EC_{50} concentration resulting in 50% of maximum effect, K_a absorption rate constant, *LLOQ* lower limit of quantification, *MRT* mean residence time, *PD* pharmacodynamic, *PK* pharmacokinetic, *PMN* polymorphonuclear cell, *RBC*, red blood cell, T_{max} time to maximum plasma concentration, *V* central volume of distribution.

Online Resource 4 Goodness of fit plots for PK model diagnostics, showing (**a**) observations versus population (top left) and individual predictions (top right), individual weighted residuals versus individual predictions (middle left), and conditional weighted residuals versus time after dose (middle right); and (**b**) conditional weighted residuals versus body weight.

a

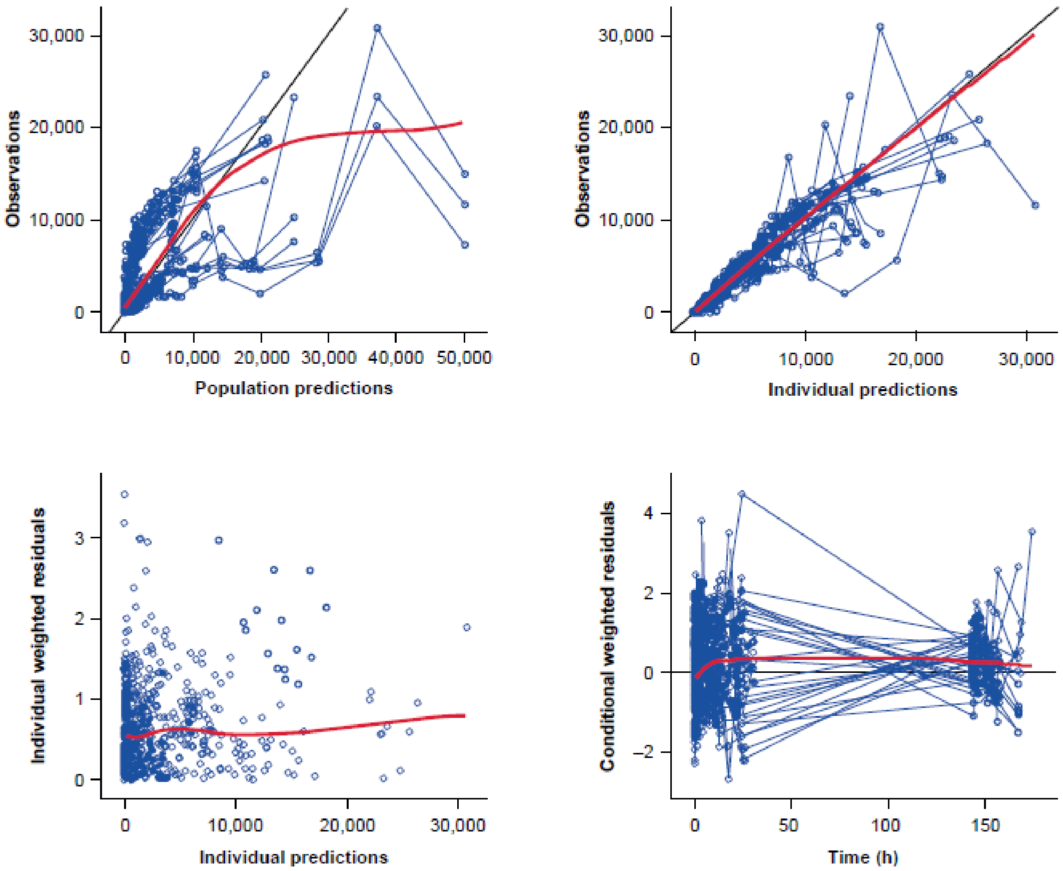

b

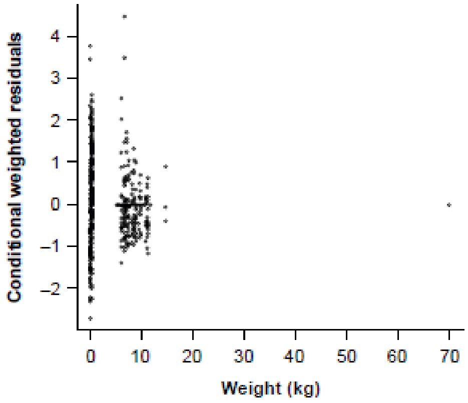

Online Resource 5 PD model diagnostics. (**a**) Visual predictive checks of polymorphonuclear cells versus time, including data from mouse (Species 1), rat (Species 2), and dog (Species 3). Blue circles represent observed values; solid red line represents median observed values binned by time; dashed red lines represent the upper and lower limits of the 95% CI interval for the observed data; red and blue shaded areas represent the 95% CI for the median, and upper and lower limits of the 95% CI interval, for the simulated data, respectively. (**b**) Condition weighted residuals versus weight plot.

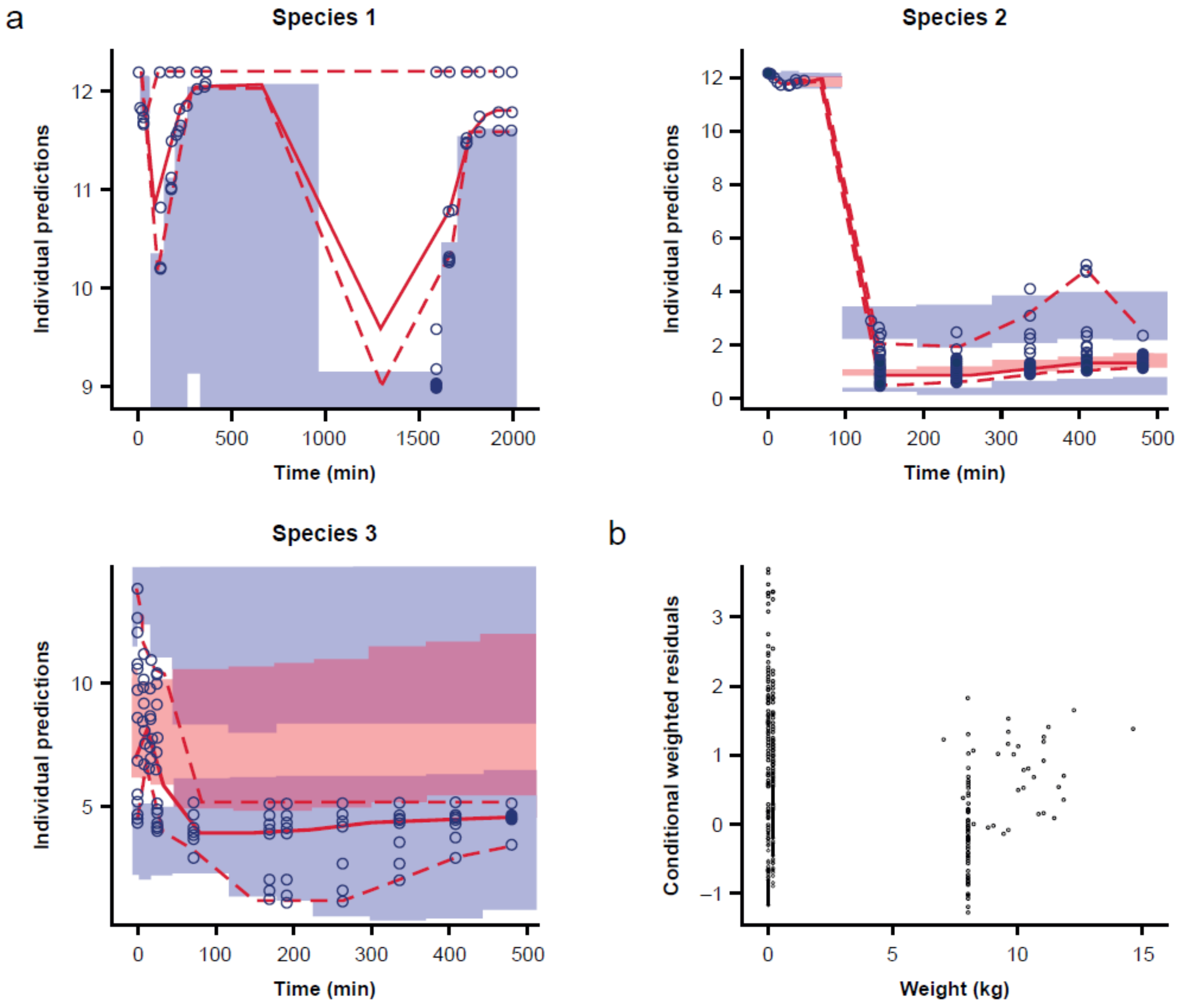

Online Resource 6 Summary of key PK parameters in Studies G1T28-1-01, G1T28-02 (Part 1), and G1T28-03 (Part 1)

Study	Cohort	C_{max} (ng/mL)	T_{max} (h)	$T_{1/2}$ (h)	$AUC_{0\text{-inf}}$ (ng h/mL)	CL (L/h)	Vz (L)
G1T28-1-01	Trilaciclib 6 mg/m^2 (*n* = 3)	50.4	0.25	5.32	66.6	188	1459
		(42.7–60.5)	(0.25–0.47)	(3.90–8.17)	(57.1–86.6)	(152–212)	(1130–1798)
	Trilaciclib 12 mg/m^2 (*n* = 3)	119	0.47	7.62	143	162	1773
		(87.4–143)	(0.25–0.47)	(6.96–8.21)	(119–182)	(120–192)	(1422–2146)
	Trilaciclib 24 mg/m^2 (*n* = 3)	255	0.47	9.28	279	176	2310
		(177–334)	(0.25–0.47)	(7.35–13.8)	(220–392)	(125–203)	(2137–2493)
	Trilaciclib 49 mg/m^2 (*n* = 3)	224	0.47	12.9	518	176	3430
		(217–228)	(0.25–0.48)	(9.96–18.5)	(472–544)	(168–186)	(2495–4960)
	Trilaciclib 96 mg/m^2 (*n* = 6)	910	0.37	14.7	1341	142	3054
		(654–1320)	(0.25–0.48)	(10.2–18.7)	(1108–1614)	(109–170)	(2300–4374)
	Trilaciclib 192 mg/m^2 (*n* = 6)	1969	0.47	14.5	3106	117	2461
		(1460–2930)	(0.25–0.47)	(13.0–16.5)	(2495–3869)	(90–145)	(1844–3011)
	BED cohort trilaciclib 192 mg/m^2 (*n* = 12)	1705	0.47	14.5	2991	126	2644
		(885–3280)	(0.25–0.48)	(11.9–17.3)	(2379–3762)	(103–145)	(1885–3122)
	Both 192 mg/m^2 cohorts (*n* = 18)	1789	0.47	14.5	3029	123	2583
		(885–3280)	(0.25–0.48)	(11.9–17.3)	(2379–3869)	(90–145)	(1844–3122)
G1T28-02	Trilaciclib 200 mg/m^2, Cycle 1 Day 1 (*n* = 8)	1240	NC	7.87	2560	150	1700
		(234–2170)	(0.45–0.98)	(5.70–10.6)	(1390–3730)	(107–240)	(1610–2790)
	Trilaciclib 200 mg/m^2, Cycle 1 Day 3 (*n* = 8)	1620	NC	9.12	3110[a]	134	1770
		(312–3360)	(0.47–1.00)	(6.99–11.0)	(2090–3910)	(94–186)	(1230–2580)
	Trilaciclib 240 mg/m^2, Cycle 1 Day 1 (*n* = 1)	1570	NC	2.91	2280	231	967
		(NC–NC)	(NC–NC)	(NC–NC)	(NC–NC)	(NC–NC)	(NC–NC)
	Trilaciclib 240 mg/m^2, Cycle 1 Day 3 (*n* = 1)	2260	NC	4.03	2960[a]	180	1050
		(NC–NC)	(NC–NC)	(NC–NC)	(NC–NC)	(NC–NC)	(NC–NC)
G1T28-03	Trilaciclib 200 mg/m^2, Cycle 1 Day 1 (*n* = 10)	1200	NC	6.92	2410	165	1650
		(660–2610)	(0.42–0.67)	(5.60–9.20)	(1700–3000)	(112–259)	(1220–2390)

Trilaciclib 200 mg/m², Cycle 1 Day 4 (*n* = 9)	1060 (520–2340)	NC (0.38–0.58)	8.57 (6.85–11.0)	2320[a] (1710–3110)	171 (124–258)	2120 (1280–3550)
Trilaciclib 240 mg/m², Cycle 1 Day 1 (*n* = 14)	1270 (410–12100)	NC (0.40–0.70)	6.79 (5.01–10.5)	2910 (1610–7670)	145 (61–25)	1430 (470–2520)
Trilaciclib 240 mg/m², Cycle 1 Day 4 (*n* = 15)	1220 (524–11500)	NC (0.38–1.50)	8.09 (6.35–9.98)	2880[a] (1740–6350)	148 (61–291)	1730 (689–2960)
Trilaciclib 280 mg/m², Cycle 1 Day 1 (*n* = 7)	1110 (679–2280)	NC (0.42–0.62)	7.38 (6.43–9.53)	3490 (2530–4750)	161 (131–219)	1720 (1270–2430)
Trilaciclib 280 mg/m², Cycle 1 Day 4 (*n* = 7)	2220 (794–7300)	NC (0.42–0.55)	8.54 (7.46–9.92)	4520[a] (2900–7010)	125 (84–176)	1530 (987–2520)

Values shown are mean (range) for G1T28-1-01 and G1T28-02, and geometric mean (range) for G1T28-03

[a]AUC_{tau} (area under the plasma concentration-time curve for the dosing interval)

$AUC_{0\text{-}inf}$ area under the plasma concentration-time curve from time 0 to infinity, *BED* biologically effective dose, *CL* clearance, C_{max} maximum plasma concentration, *NC* not calculated, *PK* pharmacokinetic, $T_{1/2}$ terminal half-life, T_{max} time to maximum plasma concentration, V_z volume of distribution during the terminal phase

Online Resource 7 Summary of myelosuppression endpoints in Part 1 of Study G1T28-02 (Full Analysis Set, all randomized patients who received at least one dose of any study drug) and Part 1 of Study G1T28-03 (Intent to Treat Analysis Set, all enrolled patients who received at least one dose of any study drug)

Endpoint	G1T28-02			G1T28-03						
	Trilaciclib 200 mg/m^2 (*n* = 10)	Trilaciclib 240 mg/m^2 (*n* = 9)	Trilaciclib total (*n* = 19)	Trilaciclib 200 mg/m^2 + topotecan 1.5 mg/mg^2 (*n* = 2)	Trilaciclib 200 mg/m^2 + topotecan 1.25 mg/mg^2 (*n* = 3)	Trilaciclib 200 mg/m^2 + topotecan 0.75 mg/mg^2 (*n* = 4)	Trilaciclib 240 mg/m^2 + topotecan 0.75 mg/mg^2 (*n* = 8)	Trilaciclib 280 mg/m^2 + topotecan 0.75 mg/mg^2 (*n* = 7)	Trilaciclib 240 mg/m^2 + topotecan 1.00 mg/mg^2 (*n* = 8)	Total (*n* = 32)
DSN in Cycle 1, days, mean (SD)	6 (5.7)[a]	–	6 (5.7)[a]	14 (1.4)	8 (6.8)	0 (0.0)	0 (0.0)	2 (3.6)	3 (5.5)	3 (5.1)
Occurrence of SN, *n* (%)	4 (40)	0	4 (40)	2 (100)	2 (66.7)	1 (25.0)	0	2 (28.6)	3 (37.5)	10 (31.3)
Occurrence of RBC transfusions on/after Week 5, *n* (%)	4 (40.0)	1 (11.1)	5 (26.3)	2 (100)	1 (33.3)	2 (50.0)	1 (12.5)	1 (14.3)	2 (25.0)	9 (28.1)
Occurrence of G-CSF administration, *n* (%)	5 (50.0)	3 (33.3)	8 (42.1)	2 (100)	2 (66.7)	1 (25.0)	4 (50.0)	2 (28.6)	3 (37.5)	14 (43.8)
Occurrence of platelet transfusions, *n* (%)	1 (10.0)	0	1 (5.3)	0	1 (33.3)	0	0	1 (14.3)	0	2 (6.3)
MAHE, event rate (per week)	–	–	–	0.403	0.156	0.102	0.037	0.085	0.073	0.107
Time (months) to first MAHE, median (95% CI)	2.6 (0.3–NE)	3.0 (0.9–NE)	2.6 (0.9–NE)	–	–	–	–	–	–	–
Patients with any event for MAHE, *n* (%)	6 (60.0)	6 (66.7)	12 (63.2)	–	–	–	–	–	–	–
Occurrence of Grade 3 or 4 hematologic laboratory abnormalities, *n* (%)	4 (40.0)	1 (11.1)	5 (26.3)	2 (100.0)	3 (100.0)	4 (100.0)	7 (87.5)	7 (100.0)	7 (87.5)	30 (93.8)
Mean ANC Nadir in Cycle 1 ($\times 10^9$/L)	1.198	1.653	1.414	0.20 (0.000)	0.47 (0.551)	0.98 (0.330)	1.31 (0.650)	0.86 (0.594)	0.63 (0.341)	–

Occurrence of Grade 3 or 4 decreased hemoglobin laboratory values, *n* (%)	–	–	–	2 (100)	1 (33.3)	2 (50.0)	3 (37.5)	1 (14.3)	3 (37.5)	12 (37.5)
Occurrence of Grade 3 or 4 decreased platelet count values, *n* (%)	–	–	–	2 (100)	1 (33.3)	2 (50.0)	0	2 (28.6)	1 (12.5)	8 (25.0)
Occurrence of ESA administrations, *n* (%)	2 (20.0)	0	2 (10.5)	2 (100)	1 (33.3)	0	1 (12.5)	0	1 (12.5)	5 (15.6)
Occurrence of IV antibiotic use, *n* (%)	4 (40.0)	1 (11.1)	5 (26.3)	2 (100)	1 (33.3)	1 (25.0)	1 (12.5)	2 (28.6)	1 (12.5)	8 (25.0)
Occurrence of infection SAEs, *n* (%)	2 (20.0)	1 (11.1)	3 (15.8)	2 (100)	1 (33.3)	1 (25.0)	0	0	1 (12.5)	5 (15.6)
Occurrence of pulmonary infection SAEs *n* (%)	1 (10.0)	0	1 (5.3)	1 (50.0)	1 (33.3)	1 (25.0)	0	0	1 (12.5)	4 (12.5)
Occurrence of FN, *n* (%)	0	0	0	0	0	0	0	1 (14.3)	0	1 (3.1)

[a]Median (SD); evaluated for the period from Cycle 1 Day 1 to date of the last assessment/contact date prior to or on the day of study completion/discontinuation, or lost to follow-up, whichever was earlier

ANC absolute neutrophil count, *CI* confidence interval, *DSN* duration of severe neutropenia, *ESA* erythropoietin-stimulating agent, *FN* febrile neutropenia, *G-CSF* granulocyte colony–stimulating factor, *IV* intravenous, *MAHE* major adverse hematologic event, *NE* not evaluable, *RBC* red blood cell, *SAE* serious adverse event, *SD* standard deviation, *SN* severe neutropenia
